# High-field magnetic resonance imaging of structural alterations in first-episode, drug-naive patients with major depressive disorder

**DOI:** 10.1038/tp.2016.209

**Published:** 2016-11-08

**Authors:** Z Chen, W Peng, H Sun, W Kuang, W Li, Z Jia, Q Gong

**Affiliations:** 1 Department of Radiology, Huaxi MR Research Center (HMRRC), West China Hospital of Sichuan University, Chengdu, China; 2 Department of Psychiatry, West China Hospital of Sichuan University, Chengdu, China; 3 Department of Nuclear Medicine, West China Hospital of Sichuan University, Chengdu, China; 4 Department of Psychology, School of Public Administration, Sichuan University, Chengdu, China

## Abstract

Previous structural imaging studies have found evidence of brain morphometric changes in patients with major depressive disorder (MDD), but these studies rarely excluded compounding effects of certain important factors, such as medications and long duration of illnesses. Furthermore, the neurobiological mechanism of the macroscopic findings of structural alterations in MDD patients remains unclear. In this study, we utilized magnetization transfer imaging, a quantitative measure of the macromolecular structural integrity of brain tissue, to identify biophysical alterations, which are represented by a magnetization transfer ratio (MTR), in MDD patients. To ascertain whether MTR changes occur independent of volume loss, we also conduct voxel-based morphometry (VBM) analysis. The participants included 27 first-episode, drug-naive MDD patients and 28 healthy controls matched for age and gender. Whole-brain voxel-based analysis was used to compare MTR and gray matter volume across groups and to analyse correlations between MTR and age, symptom severity, and illness duration. The patients exhibited significantly lower MTR in the left superior parietal lobule and left middle occipital gyrus compared with healthy controls, which may be related to the attentional and cognitive dysfunction in MDD patients. The VBM analysis revealed significantly increased gray matter volume in right postcentral gyrus in MDD patients. These findings in first-episode, drug-naive MDD patients may reflect microstructural gray matter changes in the parietal and occipital cortices close to illness onset that existed before volume loss, and thus potentially provide important new insight into the early neurobiology of depression.

## Introduction

As one of the leading worldwide causes of disability, major depressive disorder (MDD) is characterized by persistent, pervasive feelings of sadness, guilt and worthlessness, and often results in an increased risk of suicide.^1^ MDD causes significant individual suffering and impairs social and occupational functioning, thus resulting in a major public health and economic burden. *In vivo* studies of the brains of MDD patients serve as an important strategy for learning about the neurobiology of depression and are helpful for developing effective therapies.

In recent decades, numerous structural magnetic resonance imaging (MRI) studies have sought to identify the key brain areas involved in the pathogenesis of depressive symptoms. Voxel-based morphometry (VBM) studies have reported that MDD is associated with widespread local abnormalities in many brain regions, such as the frontal gyrus,^2, 3, 4^ insula,^4^ temporal lobes,^4^ anterior cingulate cortex,^3, 4, 5^ striatum^6^ and hippocampus.^7^ Larger volumes of white matter in the inferior parietal lobule^8^ and periventricular^9^ regions and deep or subcortical^10^ white matter hyperintensity have also been reported. However, these reports are inconsistent in detail, probably because of potential confounders associated with medications and/or long duration of illnesses. Thus, studies of first-episode, drug-naive MDD are a starting point for assessing brain structure before it is influenced by potential confounders and thereby provide core information relevant to models of pathogenesis.^11^

The neurobiological mechanism of the macroscopic structural alterations in MDD patients remains unclear. A post-mortem study has demonstrated reduced levels of the metabotropic glutamate receptor 5 monomer protein in the prefrontal cortex in cases of depression.^12^ MDD patients have also showed reduced density of neuronal cell bodies with large cell body size in cortical layers 2 through 5 of the orbitofrontal cortex (OFC), and in layers 2, 3 and 6 of the dorsolateral prefrontal cortex (dlPFC), concurrent with an increased density of small body size neurons in layer 3 (OFC) and layers 3 and 6 (dlPFC).^13, 14^ Observations of reduced glial cell density in the dlPFC and anterior cingulate cortex in the post-mortem brain of depressed patients provide further evidence of glial changes in patients with MDD.^15, 16^ However, it is difficult to characterize the histopathological changes *in vivo* that underlie the macroscopic abnormalities. Techniques with sufficient neuropathological sensitivity to relatively subtle macromolecules are needed.

Magnetization transfer imaging (MTI) is a MR technique with the potential for providing more neuropathological information *in vivo* to subtle or early neuropathological changes than volumetric MRI.^17, 18^ By utilizing the magnetization exchange between the spins of free water and water bound to macromolecules, MTI creates a contrast between tissues, namely, magnetization transfer ratio (MTR) map. The MTR measures the efficiency of these exchange phenomena, which depends on biophysical integrity of macromolecular protein pools and their local microenvironment.^19^ MTI has been used to characterize the status of macromolecular proteins in several clinical brain disorders including depression,^20, 21, 22, 23^ schizophrenia^24^ and multiple sclerosis, even when a conventional MRI is negative.^25, 26^ Previous MTI studies of depression have reported lower MTR in the corpus callosum, caudate nucleus, putamen, and the occipital white matter in late-life depressed patients,^21^ and lower MTR in the head of the caudate nucleus in both MDD patients^22^ and MDD patients with diabetes.^27^ Treatment-resistant depressed patients have been reported to show reduced MTR in the anterior cingulate cortex, insula, caudate tail and amygdala-parahippocampal areas compared with healthy controls.^20^

However, these studies included patients taking antidepressants or patients who were free of antidepressants for at least 2 weeks. Furthermore, most of these studies used only region-of-interest (ROI) methods that focused on specific brain regions. In this study, we use both whole brain and ROI MTI approaches to explore and further characterize the neuropathological abnormalities *in vivo* in first-episode, drug-naive MDD patients relative to healthy controls without the confounding effects of antidepressant therapy. To ascertain whether MTR changes occurs independent of volume loss, we also conduct VBM analysis.

## Materials and methods

### Subjects

We recruited patients with 27 MDD from the Department of Psychiatry at West China Hospital of Sichuan University. All patients were assessed by an experienced psychiatrist and determined to meet the Diagnostic and Statistical Manual of Mental Disorders-IV (DSM-IV) criteria for MDD.^28^ All patients also met the following inclusion criteria: (1) were between 18 and 60 years; (2) were experiencing first-episode depression; and (3) had a 17-item Hamilton Rating Scale for Depression (HRSD)^29^ total score of 18 or higher on the day of their scan. Exclusion criteria included the following: (1) currently taking or had previously taken antidepressant or anti-psychotic drugs; (2) currently taking psychotropic medications, including benzodiazepines, hypnotics or anticonvulsant agents; (3) a history of electroconvulsive therapy; (4) contraindications to the MRI scan; (5) any psychotic disorders other than MDD; and (6) significant medical or neurological illness, including any history of significant head trauma with the loss of consciousness.

We recruited 28 healthy comparison subjects from the local area by poster advertisement who were assessed using the Structured Clinical Interview for the DSM-IV. Exclusion criteria included the same medical and psychiatric factors used to recruit MDD patients, as well as any DSM-IV Axis I disorder or known history of significant psychiatric illness. All participants were of Chinese Han nationality.

The West China Hospital Clinical Trials and Biomedical Ethics Committee of Sichuan University approved the study protocol (registration no. 84). The experiment was performed in accordance with the approved guidelines, and written informed consent was obtained from all participants.

### MRI Acquisition

Images were acquired using a 3.0 Tesla General Electric magnetic resonance scanner (EXCITE, General Electric Medical Systems, Milwaukee, WI, USA). Participants were fitted with soft earplugs that were positioned comfortably in the coil, and they were then instructed to relax and remain still. Head motion was minimized using foam pads. Whole-brain MT images were acquired using a three-dimensional fast, low-angle shot sequence. One acquisition was performed with, and another without, the magnetization saturation pulse at 1.5 kHz off-resonance. In this way, MT- and non-MT-weighted images were generated separately. The other sequence parameters were as follows: repetition time/echo time (TR/TE)=37/5 ms; flip angle=15° 50 contiguous axial slices with slice thickness=3 mm; field of view=24 × 24 cm^2^; and data matrix=320 × 192. High-resolution, three-dimensional, T1-weighted images were acquired using a spoiled gradient recalled sequence (repetition time=8.5 ms, echo time=3.4 ms, fractional anisotropy=12°, 156 axial slices with thickness of 1 mm, axial field of view=240 × 240 mm, data matrix=256 × 256).

### Image processing

MR images of all the subjects were first reviewed by a neuro-radiologist to ensure that there were no structural abnormalities or data quality flaws. Data processing and analyses were conducted using the statistical parametric mapping software SPM8 (Wellcome Trust Centre for Neuroimaging, London, UK; http://www.fil.ion.ucl.ac.uk/spm). For each subject, we first co-registered the MT- and non-MT-weighted images using a mutual information registration algorithm. MTR was calculated on a voxel-by-voxel basis as follows: MTR=(*M*_0_−*M*_s_)/*M*_0_ × 100, where *M*_0_ and *M*_s_ are the signal intensities without and with, respectively, the saturation pulse applied. As the non-MT images are partially T1-weighted, we directly normalized them to the MNI T1 W template and then used the transformation parameters to normalize the co-registered MTR map. The normalized non-MT images were skull-stripped using the brain extraction tool (http://www.fmrib.ox.ac.uk/fsl/bet/)^30^ and were then used as masks to remove non-brain tissues on the normalized MTR maps. Finally, MTR maps were smoothed with a Gaussian kernel of 6-mm full width at half maximum.

### Voxel-based magnetization transfer analysis

MTR maps of MDD patients were compared with those of healthy controls using two-tailed two-sample *t*-tests controlling for age and gender in SPM8 (Wellcome Trust Centre for Neuroimaging). Significance in the resulting statistical maps was set at 0.05 and corrected for multiple comparisons. The correction standard was determined by Monte Carlo simulations, with the following parameters: individual voxel *P*-value=0.01, 1000 simulations, full width at half maximum=6 mm, applied using the Resting-State fMRI Data Analysis Toolkit of the AlphaSim program (http://www.restfmri.net).^31^ In this way, a corrected significance level of *P*<0.05 was obtained for a minimum cluster size of 132 voxels. To quantify changes in the affected regions, MTR values were extracted using a volume-of-interest approach in SPM. We conducted correlation analyses between the average regional values in these regions and the patients' age, HRSD score and illness duration.

### ROI analysis

**Figure 1:**
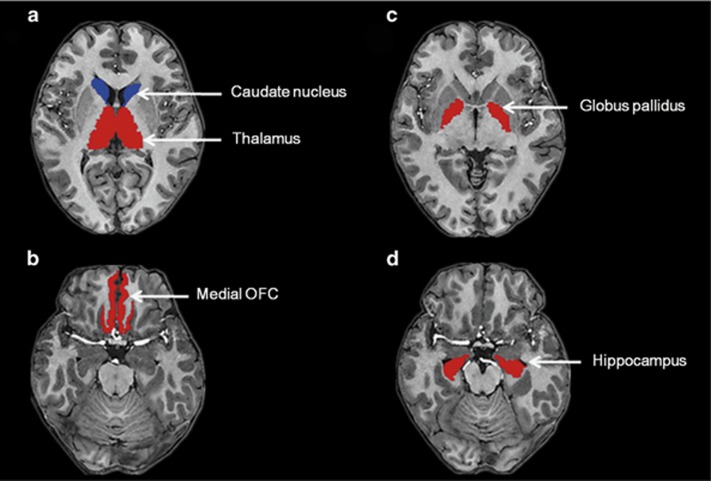
Regions of interest of (**a**) caudate nucleus, thalamus; (**b**) medial orbitofrontal cortex (OFC); (**c**) globus pallidus; (**d**) hippocampus for magnetization transfer ratio analysis.

We were also interested in determining the MTR changes of specific brain regions, such as the bilateral medial OFC, hippocampus, caudate nucleus, globus pallidus and thalamus, as they have been frequently reported in studies of depression.^32, 33, 34^ These brain regions were selected as ROIs, and the segmentation was performed with the Freesurfer software package (http://surfer.nmr.mgh.harvard.edu/; Figure 1). In brief, we first conducted the atlas-based analysis using the Desikan–Killiany atlas^35^ to segment the T1 image of each subject into 68 cortical regions (34 per hemisphere) and 40 subcortical structures. The T1 image of each subject was then co-registered to his/her non-MT-weighted image. The resultant transformation was used to map the segmented map to the non-MT image space. Finally, we overlaid the segmented map onto the MTR images and extracted the MTR values of bilateral ROIs using ITK-SNAP (www.itksnap.org),^36^ and the MTR values of MDD patients were compared with those of healthy controls using two-tailed univariate analysis of covariance controlling for age and gender.

### VBM analysis

To identify whether morphological abnormalities could be detected in the first-episode, drug-naive MDD patients, T1W SPGR images were used to conduct VBM analysis using Exponentiated Lie algebra (DARTEL)^37^ as implemented in SPM8. Two-tailed two-sample *t*-tests controlling for age and gender were performed to compare group differences of gray matter volume between first-episode, drug-naive MDD patients and healthy controls. Significance in the resulting statistical maps was set at 0.05 and corrected for multiple comparisons using the Resting-State fMRI Data Analysis Toolkit AlphaSim utility same as in voxel-based magnetization transfer analysis.

## Results

### Demographic and clinical comparisons

Table 1 presents the demographic and clinical characteristics of the study participants. There were no significant differences between the first-episode, drug-naive MDD patients and the healthy controls in gender, age or handedness (*P*>0.05).

### Voxel-based magnetization transfer analysis

**Figure 2:**
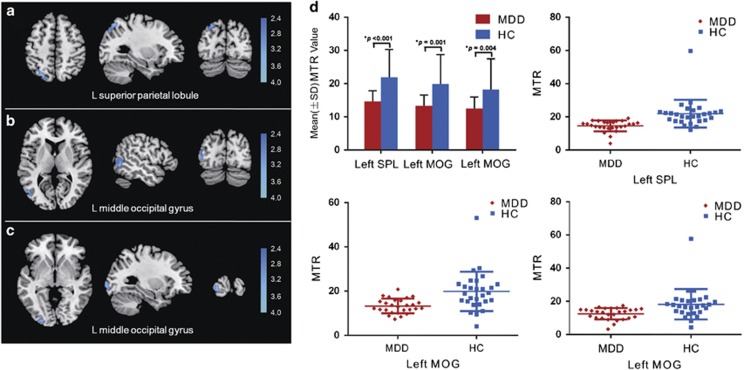
Magnetization transfer ratio differences in voxel-based analysis comparisons between patients with major depressive disorder and healthy controls. Images are presented in neurological orientations. Patients with major depressive disorder exhibited a reduced magnetization transfer ratio in (**a**) the left superior parietal lobule and (**b** and **c**) the left middle occipital gyrus relative to healthy controls. Statistical inferences were made with a voxel-level statistical threshold of *P*<0.05 (corrected). (**d**) Quantification of MTR values in the affected regions. HC, healthy control; L, left; MDD, major depressive disorder; MOG, middle occipital gyrus; MTR, magnetization transfer ratio; SPL, superior parietal lobule.

As presented in Table 2, the first-episode, drug-naive MDD group exhibited lower MTR in the left superior parietal lobule (SPL; Figure 2a) and left middle occipital gyrus (MOG; Figures 2b and c) compared with the healthy controls (*P<*0.05, corrected for multiple comparisons). No marked regional MTR increase in the MDD group compared with the controls was found. Figure 2d illustrates the changes in the MTR values in the affected regions (*t* =4.244, *P*<0.001 for left SPL; *t* =3.681, *P*=0.001 and *t* =3.014, *P*=0.004 for the two left MOG regions, respectively). No association was found between regional MTR values and patient age, HRSD scores or illness durations (*P*=0.391 for age and left SPL; *P*=0.05 and *P*=0.985 for age and two left MOG regions; *P*=0.073 for HRSD scores and left SPL; *P*=0.424 and *P*=0.507 for HRSD scores and two left MOG regions, respectively; *P*=0.143 for illness duration and left SPL; *P*=0.319 and *P*=0.8 for illness duration and two left MOG regions, respectively).

### ROI analysis

A ROI analysis revealed no significant group differences between the MDD patients and healthy controls in the MTR in bilateral medial OFC (left *t*=1.801, *P*=0.186; right *t*=0.238, *P*=0.628), hippocampus (left *t*=0.582, *P*=0.434; right *t*=0.032, *P*=0.437), caudate nucleus (left *t*=0.485, *P*=0.489; right *t*=0.178, *P*=0.675), globus pallidus (left *t*=1.198, *P*=0.465; right *t*=1.227, *P*=0.24) or thalamus (left *t*=0.114, *P*=0.737; right *t*=0.916, *P*=0.343) when controlling for age and gender (Supplementary Table 1).

### VBM analysis

**Figure 3:**
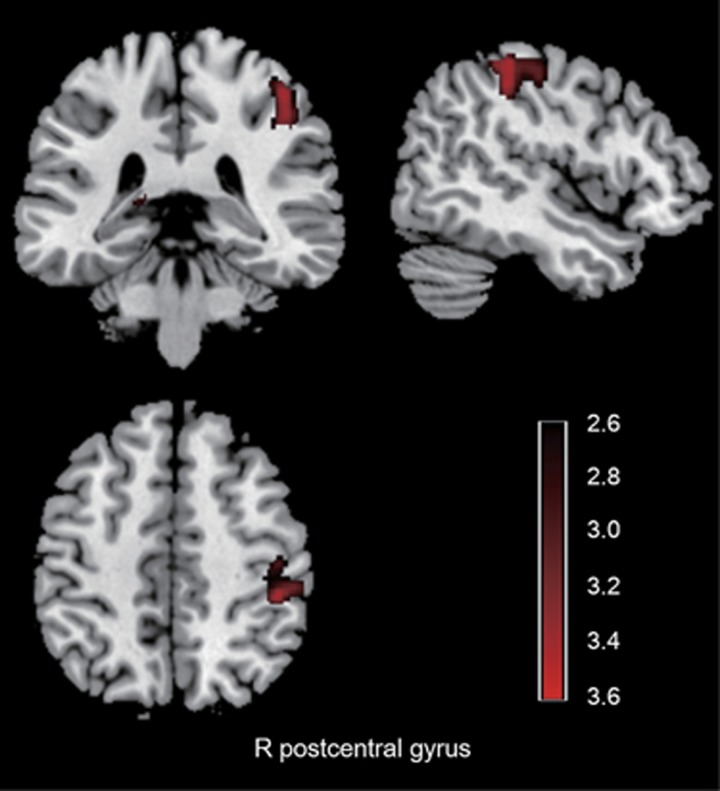
Gray matter volume differences in voxel-based analysis comparisons between patients with major depressive disorder and healthy controls. Images are presented in neurological orientations. Patients with major depressive disorder exhibited increased gray matter volume in the right postcentral gyrus compared with healthy controls. Statistical inferences were made with a voxel-level statistical threshold of *P*<0.05 (corrected). R, right.

The VBM analysis revealed increased gray matter volume in the right postcentral gyrus in first-episode, drug-naive MDD group compared with the healthy controls (*P<*0.05, corrected for multiple comparisons; Table 2; Figure 3). No marked gray matter decrease in the MDD group compared with the controls was found.

## Discussion

In the present study, we used MTI to explore the microstructural changes in first-episode, drug-naive MDD patients relative to healthy controls. The main findings are that the MDD patients exhibited lower MTR in the left superior parietal lobule and left middle occipital gyrus relative to healthy controls. To our knowledge, this is the first MTI study of first-episode, drug-naive MDD patients using both voxel-based and ROI analyses.

MTI techniques provide semi-quantitative (MTR) metrics to assess the integrity of macromolecules in the brain tissue such as myelin or cellular membranes components (proteins, cholesterol, phosphatidylcholine and galactocerebrosides).^38^ The neuropathological correlates of the MTR changes involving gray and white matter are under contention and may be related to multiple neurobiological alterations.^18, 39, 40, 41^ Reductions of MTR in gray matter are indicative of decreased size and number of neurons, or dendritic density, and may reflect abnormal cell membrane structure.^39^ In the white matter, MTR has been shown to reflect mainly demyelination, some neuro-axonal loss and, to a lesser extent, microglial activation.^42^

In this study, we found the first-episode, drug-naive MDD patients had a significantly lower MTR in the left SPL than did the healthy controls. The SPL is an important brain region of the dorsal attention network, which is hypothesized to have a key role in the top-down, or goal-driven, allocation of attention.^43^ This brain region has been consistently associated with the volitional allocation of attention to visual aspects of, and coordinated motor actions to, specific regions in extrapersonal space.^44, 45^ Previous functional MRI studies have revealed that shifting the location of attention produces a rapid, transient increase in SPL activation.^46, 47^ Impairments in attention may be due to the neuropsychological deficits that correspond to the cognitive criterion ‘impaired ability to think or concentrate' as stipulated for MDD in the DSM-IV.^28^ A structural neuroimaging study has reported a reduction in the gray matter volume in the SPL in MDD patients compared with normal subjects.^48^ Functional MRI studies have reported that first-episode, drug-naive MDD patients exhibited reduced resting-state activity in the parietal lobe,^49^ increased activation in bilateral SPL after escitalopram oxalate treatment during sad facial expression recognition^50^ and decreased functional connectivity between the dlPFC and parietal lobe in first-episode MDD patients.^51^ Furthermore, decreased glucose metabolism was also observed in the parietal cortex of depressed patients compared with that of the healthy controls.^52^ Therefore, our findings are generally consistent with these studies and further reveal that the microstructural gray matter changes in the parietal lobe may have already existed in the early stage of MDD because our group only included first-episode, drug-naive MDD patients. Together with previous findings on the parietal lobe in MDD patients, our result of lower MTR in the superior parietal lobe of MDD patients may improve our understanding of the cerebral mechanisms underlying attentional deficits in depression.

We also identified a lower MTR in the left middle occipital gyrus in first-episode, drug-naive MDD patients compared with that of healthy controls. The middle occipital gyrus is within the visual recognition network^53^ and is involved in the perception of facial emotion.^54^ Depressed patients are thought to have a negative cognitive bias in their appraisal of people or life events and are believed to be unable to carry out normal interpersonal interactions.^55^ Thus, abnormal facial emotion processing may give rise to some of the affective and social symptoms in major depression. Neuroimaging studies have suggested that the occipital cortex may be relevant in MDD. A study comparing patients with psychotic major depression, patients with non-psychotic major depression and a healthy group of individuals revealed greater right occipital activation only in the non-psychotic major depression group compared with healthy controls during a verbal working memory task, thus suggesting that increased effort is required for basic visual processing.^56^ Decreased resting-state activity in the left middle occipital gyrus has been reported in MDD patients compared with healthy controls.^57, 58^ It has also been reported that regional cerebral blood flow (rCBF) in the parieto-occipital regions was decreased in MDD patients and was significantly increased following pharmacotherapy.^59^ A magnetic resonance spectroscopy study found that gamma-aminobutyric acid (GABA) neurotransmission was altered in the occipital cortex of patients with MDD.^60^ The molecular mechanism behind the neuroimaging results may be related to the reduced number of GABAergic neurons in the occipital cortex.^61^ Consistent with our results, these findings of microstructural gray matter changes alterations in the middle occipital gyrus indicate that the visual recognition network interference may contribute to cognitive dysregulation in MDD.

Whole-brain voxel-based analysis investigates region-specific changes by spatially normalizing brain images and conducting statistical tests at each voxel, which is both reproducible and automatic. Whereas ROI analysis, usually applied in the original image space, allows one to carry out group analyses of functionally corresponding regions with high statistical power and substantially reduces the correction needed for multiple comparisons as is needed for the many thousands of voxels of a whole-brain analysis.^62^ In this study, bilateral medial OFC, hippocampus, caudate nucleus, globus pallidus and thalamus were chosen as ROIs based on previous studies and no significant difference of MTR was found between MDD patients and healthy control group that was consistent with the our whole-brain voxel-based analysis. Nonetheless, lower MTR has been demonstrated in gray matter regions in previous MTI studies of MDD.^20, 22, 63^ Two MTI studies compared the MTR value changes between MDD patients and healthy controls by manually placing ROIs in the key components of cortical–subcortical circuits—the caudate, thalamic, striatal, orbitofrontal, anterior cingulate and dorsolateral prefrontal regions, and the results showed significant group differences of MTR values in the bilateral caudate^22^ and thalamus.^63^ However, it should be noted that the MDD patients in these two studies were free of antidepressant medication at least 2 weeks, whereas patients in our study were first-episode and drug-naive. In another whole-brain MTR study, treatment-resistant depressed patients with long mean illness duration were recruited, and the results showed a reduced MTR in the anterior cingulate cortex, insula, caudate tail and amygdala-parahippocampal areas relative to healthy controls.^20^ The discrepancy between our results and those of previous studies may be mainly attributed to inter-study differences in sample size, the sociodemographic and clinical characteristics of the participants, such as the various medication statuses and the incompatible illness duration.

The VBM analysis revealed increased gray matter volume in the right postcentral gyrus in the first-episode, drug-naive MDD group compared with the healthy controls. The postcentral gyrus is the primary sensory area of the brain. The somatosensory-related cortices are considered to not only encode bodily sensations but also have a key role in using social cues to understand emotional states of others.^64^ A study has reported that the MDD patients showed reduced cerebral activation in the right postcentral gyrus than healthy controls during empathy for others pain.^65^ Similarly, another functional MRI study demonstrated that following escitalopram oxalate treatment, first-episode, treatment-naive MDD patients showed decreased brain activation in bilateral postcentral gyrus during emotion recognition.^50^ Although the underlying mechanism for increases in gray matter volume in our study is still unknown, one possible account for this effect may be the inflammatory response, as a compensatory effect in the early phase of depression.^66, 67^ It is worth mentioning that different regions were implicated when MTI and VBM methods were separately used. MTI provides information on the integrity of macromolecules in the brain tissue,^19^ whereas VBM allows for the study of the gray matter morphology. These isolated findings of MTI and VBM analyses suggested that MTR changes in occipital and parietal cortices existed beyond volume loss in the early stage of MDD which needs further longitudinal studies.

Our study has certain limitations. First, the sample size is modest. It is possible that a larger sample size will provide further insight. However, it should be noted that our patient group was a homogeneous group of first-episode MDD patients without medication. Second, MTR is very sensitive to pulse sequences and relaxation properties. This makes it difficult to accurately address the specific neurobiological causes of the identified MTR alterations. Third, as our study uses a cross-sectional design, the question arises whether these differences are a consequence or precondition of MDD. This issue can only be resolved by longitudinal design studies.

In conclusion, this study demonstrates that first-episode, drug-naive MDD patients exhibit structural abnormalities, namely, lower MTR in the left superior parietal lobule and left middle occipital gyrus and increased gray matter volume in right postcentral gyrus that largely avoided the confounding effects from medications and/or long duration of illness. The MTR changes in the parietal and occipital cortices existed beyond volume loss in the early stage of MDD. The results indicate that the attentional and cognitive dysfunction in MDD patients may be associated with microstructural gray matter changes in the parietal and occipital cortices close to the illness onset that existed before volume loss, and thus potentially provide important new insight into the early neurobiology of the depression.

## Figures and Tables

**Table 1:** Demographics and clinical characteristics of subjects

*Characteristics*	*MDD (*n=*27)*	*HC (*n=*28)*	P-*value*
Age (years), mean (s.d.)	33 (10.8)	33 (11.7)	0.951*
Gender (male/female)	13/14	14/14	0.891**
Handness (right/left)	27/0	28/0	>0.99**
Illness duration (months), mean (s.d.)	79 (86)	NA	—
Total HRSD score, mean (s.d.)	22 (3.4)	NA	—

Abbreviations: HC, healthy controls; HRSD, Hamilton Rating Scale for Depression; MDD, major depressive disorder; NA, not applicable.

* and ** indicate the *P-*values for the two-sample *t*-test and *χ*^2^-test, respectively.

**Table 2:** Regions showing differences of MTR and gray matter volume between MDD patients and healthy controls

*Regions*	*Coordinates of peak voxel in MNI space (*x, y,z*)*	*Cluster size (voxels)*	t *scores of peak voxel*^a^
*MTR analysis*			
HC>MDD			
L superior parietal lobule	−32, −74, 56	401	3.87
L middle occipital gyrus	−30, −100, 2	214	3.52
L middle occipital gyrus	−52, −76, 2	420	3.47
MDD>HC			
None			

*VBM analysis*			
MDD>HC			
R postcentral gyrus	45, −33, 56	825	3.45
HC>MDD			
None			

Abbreviations: HC, healthy controls; L, left; MDD, major depressive disorder; MNI, Montreal Neurological Institute Coordinate System or Template; MTR, magnetization transfer ratio; R, right; T, statistical value of peak voxel showing differences of MTR and gray matter volume between the two groups; VBM, voxel-based morphometry; *x*, *y*, *z*, coordinates of primary peak locations in the MNI space.

a All effects survived a voxel-wise statistical threshold (*P*<0.05), as corrected for multiple comparisons using AlphaSim program.

## References

[bib1] Jia Z, Huang X, Wu Q, Zhang T, Lui S, Zhang J et al. High-field magnetic resonance imaging of suicidality in patients with major depressive disorder. Am J Psychiatry 2010; 167: 1381–1390. 20843871 10.1176/appi.ajp.2010.09101513

[bib2] Scheuerecker J, Meisenzahl EM, Koutsouleris N, Roesner M, Schopf V, Linn J et al. Orbitofrontal volume reductions during emotion recognition in patients with major depression. J Psychiatry Neurosci 2010; 35: 311–320. 20569645 10.1503/jpn.090076 PMC2928284

[bib3] Salvadore G, Nugent AC, Lemaitre H, Luckenbaugh DA, Tinsley R, Cannon DM et al. Prefrontal cortical abnormalities in currently depressed versus currently remitted patients with major depressive disorder. NeuroImage 2011; 54: 2643–2651. 21073959 10.1016/j.neuroimage.2010.11.011 PMC3020249

[bib4] Schmaal L, Hibar DP, Samann PG, Hall GB, Baune BT, Jahanshad N et al. Cortical abnormalities in adults and adolescents with major depression based on brain scans from 20 cohorts worldwide in the ENIGMA Major Depressive Disorder Working Group. Mol Psychiatry 2016 e-pub ahead of print 3 May 2016;doi:10.1038/mp.2016.60. 10.1038/mp.2016.60 PMC5444023 27137745

[bib5] Tang Y, Wang F, Xie G, Liu J, Li L, Su L et al. Reduced ventral anterior cingulate and amygdala volumes in medication-naive females with major depressive disorder: a voxel-based morphometric magnetic resonance imaging study. Psychiatry Res 2007; 156: 83–86. 17825533 10.1016/j.pscychresns.2007.03.005

[bib6] Alexopoulos GS. Frontostriatal and limbic dysfunction in late-life depression. Am J Geriatr Psychiatry 2002; 10: 687–695. 12427577

[bib7] Zou K, Deng W, Li T, Zhang B, Jiang L, Huang C et al. Changes of brain morphometry in first-episode, drug-naive, non-late-life adult patients with major depression: an optimized voxel-based morphometry study. Biol Psychiatry 2010; 67: 186–188. 19897176 10.1016/j.biopsych.2009.09.014

[bib8] Yuan Y, Zhang Z, Bai F, Yu H, You J, Shi Y et al. Larger regional white matter volume is associated with executive function deficit in remitted geriatric depression: an optimized voxel-based morphometry study. J Affect Disord 2009; 115: 225–229. 18945494 10.1016/j.jad.2008.09.018

[bib9] Thomas AJ, O'Brien JT, Barber R, McMeekin W, Perry R. A neuropathological study of periventricular white matter hyperintensities in major depression. J Affect Disord 2003; 76: 49–54. 12943933 10.1016/s0165-0327(02)00064-2

[bib10] Dalby RB, Chakravarty MM, Ahdidan J, Sorensen L, Frandsen J, Jonsdottir KY et al. Localization of white-matter lesions and effect of vascular risk factors in late-onset major depression. Psychol Med 2010; 40: 1389–1399. 19895719 10.1017/S0033291709991656

[bib11] Agarwal N, Port JD, Bazzocchi M, Renshaw PF. Update on the use of MR for assessment and diagnosis of psychiatric diseases. Radiology 2010; 255: 23–41. 20308442 10.1148/radiol.09090339

[bib12] Deschwanden A, Karolewicz B, Feyissa AM, Treyer V, Ametamey SM, Johayem A et al. Reduced metabotropic glutamate receptor 5 density in major depression determined by [(11)C]ABP688 PET and postmortem study. Am J Psychiatry 2011; 168: 727–734. 21498461 10.1176/appi.ajp.2011.09111607 PMC3129412

[bib13] Rajkowska G, Miguel-Hidalgo JJ, Wei J, Dilley G, Pittman SD, Meltzer HY et al. Morphometric evidence for neuronal and glial prefrontal cell pathology in major depression. Biol Psychiatry 1999; 45: 1085–1098. 10331101 10.1016/s0006-3223(99)00041-4

[bib14] Rajkowska G. Postmortem studies in mood disorders indicate altered numbers of neurons and glial cells. Biol Psychiatry 2000; 48: 766–777. 11063973 10.1016/s0006-3223(00)00950-1

[bib15] Cotter D, Mackay D, Landau S, Kerwin R, Everall I. Reduced glial cell density and neuronal size in the anterior cingulate cortex in major depressive disorder. Arch Gen Psychiatry 2001; 58: 545–553. 11386983 10.1001/archpsyc.58.6.545

[bib16] Cotter D, Mackay D, Chana G, Beasley C, Landau S, Everall IP. Reduced neuronal size and glial cell density in area 9 of the dorsolateral prefrontal cortex in subjects with major depressive disorder. Cereb Cortex 2002; 12: 386–394. 11884354 10.1093/cercor/12.4.386

[bib17] Foong J, Symms MR, Barker GJ, Maier M, Woermann FG, Miller DH et al. Neuropathological abnormalities in schizophrenia: evidence from magnetization transfer imaging. Brain 2001; 124: 882–892. 11335691 10.1093/brain/124.5.882

[bib18] Bagary MS, Symms MR, Barker GJ, Mutsatsa SH, Joyce EM, Ron MA. Gray and white matter brain abnormalities in first-episode schizophrenia inferred from magnetization transfer imaging. Arch Gen Psychiatry 2003; 60: 779–788. 12912761 10.1001/archpsyc.60.8.779

[bib19] Wolff SD, Balaban RS. Magnetization transfer contrast (MTC) and tissue water proton relaxation *in vivo*. Magn Reson Med 1989; 10: 135–144. 2547135 10.1002/mrm.1910100113

[bib20] Zhang TJ, Wu QZ, Huang XQ, Sun XL, Zou K, Lui S et al. Magnetization transfer imaging reveals the brain deficit in patients with treatment-refractory depression. J Affect Disord 2009; 117: 157–161. 19211150 10.1016/j.jad.2009.01.003

[bib21] Kumar A, Gupta RC, Albert Thomas M, Alger J, Wyckoff N, Hwang S. Biophysical changes in normal-appearing white matter and subcortical nuclei in late-life major depression detected using magnetization transfer. Psychiatry Res 2004; 130: 131–140. 15033183 10.1016/j.pscychresns.2003.12.002

[bib22] Kumar A, Yang S, Ajilore O, Wu M, Charlton R, Lamar M. Subcortical biophysical abnormalities in patients with mood disorders. Mol Psychiatry 2014; 19: 710–716. 23877833 10.1038/mp.2013.84 PMC4159940

[bib23] Chen Z, Zhang H, Jia Z, Zhong J, Huang X, Du M et al. Magnetization transfer imaging of suicidal patients with major depressive disorder. Sci Rep 2015; 5: 9670. 25853872 10.1038/srep09670 PMC4389668

[bib24] Kubicki M, Park H, Westin CF, Nestor PG, Mulkern RV, Maier SE et al. DTI and MTR abnormalities in schizophrenia: analysis of white matter integrity. NeuroImage 2005; 26: 1109–1118. 15878290 10.1016/j.neuroimage.2005.03.026 PMC2768051

[bib25] Ge Y, Grossman RI, Udupa JK, Babb JS, Mannon LJ, McGowan JC. Magnetization transfer ratio histogram analysis of normal-appearing gray matter and normal-appearing white matter in multiple sclerosis. J Comput Assist Tomogr 2002; 26: 62–68. 11801905 10.1097/00004728-200201000-00009

[bib26] Liu Z, Pardini M, Yaldizli O, Sethi V, Muhlert N, Wheeler-Kingshott CA et al. Magnetization transfer ratio measures in normal-appearing white matter show periventricular gradient abnormalities in multiple sclerosis. Brain 2015; 138: 1239–1246. 25823475 10.1093/brain/awv065 PMC5963416

[bib27] Kumar A, Gupta R, Thomas A, Ajilore O, Hellemann G. Focal subcortical biophysical abnormalities in patients diagnosed with type 2 diabetes and depression. Arch Gen Psychiatry 2009; 66: 324–330. 19255382 10.1001/archgenpsychiatry.2008.548

[bib28] First M, Gibbon M, Spitzer R, Williams J, Benjamin L. Structured Clinical Interview for DSM-IV Axis I Disorders-Clinician Version (SCID-CV). American Psychiatric Publishing: Washington, DC, USA, 1997.

[bib29] Hamilton M. Development of a rating scale for primary depressive illness. Br J Soc Clin Psychol 1967; 6: 278–296. 6080235 10.1111/j.2044-8260.1967.tb00530.x

[bib30] Smith SM. Fast robust automated brain extraction. Hum Brain Mapp 2002; 17: 143–155. 12391568 10.1002/hbm.10062 PMC6871816

[bib31] Song XW, Dong ZY, Long XY, Li SF, Zuo XN, Zhu CZ et al. REST: a toolkit for resting-state functional magnetic resonance imaging data processing. PLoS One 2011; 6: e25031. 21949842 10.1371/journal.pone.0025031 PMC3176805

[bib32] Sheline YI. Neuroimaging studies of mood disorder effects on the brain. Biol Psychiatry 2003; 54: 338–352. 12893109 10.1016/s0006-3223(03)00347-0

[bib33] Drevets WC, Price JL, Furey ML. Brain structural and functional abnormalities in mood disorders: implications for neurocircuitry models of depression. Brain Struct Funct 2008; 213: 93–118. 18704495 10.1007/s00429-008-0189-x PMC2522333

[bib34] Graham J, Salimi-Khorshidi G, Hagan C, Walsh N, Goodyer I, Lennox B et al. Meta-analytic evidence for neuroimaging models of depression: state or trait? J Affect Disord 2013; 151: 423–431. 23890584 10.1016/j.jad.2013.07.002

[bib35] Desikan RS, Segonne F, Fischl B, Quinn BT, Dickerson BC, Blacker D et al. An automated labeling system for subdividing the human cerebral cortex on MRI scans into gyral based regions of interest. NeuroImage 2006; 31: 968–980. 16530430 10.1016/j.neuroimage.2006.01.021

[bib36] Yushkevich PA, Piven J, Hazlett HC, Smith RG, Ho S, Gee JC et al. User-guided 3D active contour segmentation of anatomical structures: significantly improved efficiency and reliability. NeuroImage 2006; 31: 1116–1128. 16545965 10.1016/j.neuroimage.2006.01.015

[bib37] Ashburner J. A fast diffeomorphic image registration algorithm. NeuroImage 2007; 38: 95–113. 17761438 10.1016/j.neuroimage.2007.07.007

[bib38] Barkovich AJ. Concepts of myelin and myelination in neuroradiology. AJNR Am J Neuroradiol 2000; 21: 1099–1109. 10871022 PMC7973874

[bib39] Khaleeli Z, Altmann DR, Cercignani M, Ciccarelli O, Miller DH, Thompson AJ. Magnetization transfer ratio in gray matter: a potential surrogate marker for progression in early primary progressive multiple sclerosis. Arch Neurol 2008; 65: 1454–1459. 19001163 10.1001/archneur.65.11.1454

[bib40] Audoin B, Davies G, Rashid W, Fisniku L, Thompson AJ, Miller DH. Voxel-based analysis of grey matter magnetization transfer ratio maps in early relapsing remitting multiple sclerosis. Mult Scler 2007; 13: 483–489. 17463071 10.1177/1352458506070450

[bib41] Derakhshan M, Caramanos Z, Narayanan S, Arnold DL, Louis Collins D. Surface-based analysis reveals regions of reduced cortical magnetization transfer ratio in patients with multiple sclerosis: a proposed method for imaging subpial demyelination. Hum Brain Mapp 2014; 35: 3402–3413. 24356893 10.1002/hbm.22410 PMC6869281

[bib42] Moll NM, Rietsch AM, Thomas S, Ransohoff AJ, Lee JC, Fox R et al. Multiple sclerosis normal-appearing white matter: pathology-imaging correlations. Ann Neurol 2011; 70: 764–773. 22162059 10.1002/ana.22521 PMC3241216

[bib43] Corbetta M, Shulman GL. Control of goal-directed and stimulus-driven attention in the brain. Nat Rev Neurosci 2002; 3: 201–215. 11994752 10.1038/nrn755

[bib44] Pessoa L, Kastner S, Ungerleider LG. Neuroimaging studies of attention: from modulation of sensory processing to top-down control. J Neurosci 2003; 23: 3990–3998. 12764083 10.1523/JNEUROSCI.23-10-03990.2003 PMC6741071

[bib45] Culham JC, Kanwisher NG. Neuroimaging of cognitive functions in human parietal cortex. Curr Opin Neurobiol 2001; 11: 157–163. 11301234 10.1016/s0959-4388(00)00191-4

[bib46] Yantis S, Schwarzbach J, Serences JT, Carlson RL, Steinmetz MA, Pekar JJ et al. Transient neural activity in human parietal cortex during spatial attention shifts. Nat Neurosci 2002; 5: 995–1002. 12219097 10.1038/nn921

[bib47] Yantis S, Serences JT. Cortical mechanisms of space-based and object-based attentional control. Curr Opin Neurobiol 2003; 13: 187–193. 12744972 10.1016/s0959-4388(03)00033-3

[bib48] Inkster B, Rao AW, Ridler K, Nichols TE, Saemann PG, Auer DP et al. Structural brain changes in patients with recurrent major depressive disorder presenting with anxiety symptoms. J Neuroimaging 2011; 21: 375–382. 20977527 10.1111/j.1552-6569.2010.00515.x

[bib49] Wang L, Li K, Zhang Q, Zeng Y, Dai W, Su Y et al. Short-term effects of escitalopram on regional brain function in first-episode drug-naive patients with major depressive disorder assessed by resting-state functional magnetic resonance imaging. Psychol Med 2014; 44: 1417–1426. 23942213 10.1017/S0033291713002031

[bib50] Jiang W, Yin Z, Pang Y, Wu F, Kong L, Xu K. Brain functional changes in facial expression recognition in patients with major depressive disorder before and after antidepressant treatment: A functional magnetic resonance imaging study. Neural Regen Res 2012; 7: 1151–1157. 25722708 10.3969/j.issn.1673-5374.2012.15.005 PMC4340032

[bib51] Ye T, Peng J, Nie B, Gao J, Liu J, Li Y et al. Altered functional connectivity of the dorsolateral prefrontal cortex in first-episode patients with major depressive disorder. Eur J Radiol 2012; 81: 4035–4040. 22939367 10.1016/j.ejrad.2011.04.058

[bib52] Biver F, Goldman S, Delvenne V, Luxen A, De Maertelaer V, Hubain P et al. Frontal and parietal metabolic disturbances in unipolar depression. Biol Psychiatry 1994; 36: 381–388. 7803599 10.1016/0006-3223(94)91213-0

[bib53] Tao H, Guo S, Ge T, Kendrick KM, Xue Z, Liu Z et al. Depression uncouples brain hate circuit. Mol Psychiatry 2013; 18: 101–111. 21968929 10.1038/mp.2011.127 PMC3526729

[bib54] Fusar-Poli P, Placentino A, Carletti F, Landi P, Allen P, Surguladze S et al. Functional atlas of emotional faces processing: a voxel-based meta-analysis of 105 functional magnetic resonance imaging studies. J Psychiatry Neurosci 2009; 34: 418–432. 19949718 PMC2783433

[bib55] Coyne JC. Depression and the response of others. J Abnorm Psychol 1976; 85: 186–193. 1254779 10.1037//0021-843x.85.2.186

[bib56] Garrett A, Kelly R, Gomez R, Keller J, Schatzberg AF, Reiss AL. Aberrant brain activation during a working memory task in psychotic major depression. Am J Psychiatry 2011; 168: 173–182. 21078708 10.1176/appi.ajp.2010.09121718

[bib57] Guo WB, Liu F, Xue ZM, Xu XJ, Wu RR, Ma CQ et al. Alterations of the amplitude of low-frequency fluctuations in treatment-resistant and treatment-response depression: a resting-state fMRI study. Prog Neuropsychopharmacol Biol Psychiatry 2012; 37: 153–160. 22306865 10.1016/j.pnpbp.2012.01.011

[bib58] Fan T, Wu X, Yao L, Dong J. Abnormal baseline brain activity in suicidal and non-suicidal patients with major depressive disorder. Neurosci Lett 2013; 534: 35–40. 23201633 10.1016/j.neulet.2012.11.032

[bib59] Ishizaki J, Yamamoto H, Takahashi T, Takeda M, Yano M, Mimura M. Changes in regional cerebral blood flow following antidepressant treatment in late-life depression. Int J Geriatr Psychiatry 2008; 23: 805–811. 18214999 10.1002/gps.1980

[bib60] Bhagwagar Z, Wylezinska M, Jezzard P, Evans J, Ashworth F, Sule A et al. Reduction in occipital cortex gamma-aminobutyric acid concentrations in medication-free recovered unipolar depressed and bipolar subjects. Biol Psychiatry 2007; 61: 806–812. 17210135 10.1016/j.biopsych.2006.08.048

[bib61] Maciag D, Hughes J, O'Dwyer G, Pride Y, Stockmeier CA, Sanacora G et al. Reduced density of calbindin immunoreactive GABAergic neurons in the occipital cortex in major depression: relevance to neuroimaging studies. Biol Psychiatry 2010; 67: 465–470. 20004363 10.1016/j.biopsych.2009.10.027 PMC2823848

[bib62] Frost MA, Goebel R. Functionally informed cortex based alignment: an integrated approach for whole-cortex macro-anatomical and ROI-based functional alignment. NeuroImage 2013; 83: 1002–1010. 23899723 10.1016/j.neuroimage.2013.07.056

[bib63] Kumar A, Yang S, Ajilore O, Wu M, Cohen J, Lamar M et al. Biophysical changes in subcortical nuclei: the impact of diabetes and major depression. Mol Psychiatry 2016; 21: 531–536. 26169972 10.1038/mp.2015.89 PMC9795853

[bib64] Keysers C, Kaas JH, Gazzola V. Somatosensation in social perception. Nat Rev Neurosci 2010; 11: 417–428. 20445542 10.1038/nrn2833

[bib65] Fujino J, Yamasaki N, Miyata J, Kawada R, Sasaki H, Matsukawa N et al. Altered brain response to others pain in major depressive disorder. J Affect Disord 2014; 165: 170–175. 24882196 10.1016/j.jad.2014.04.058

[bib66] Dowlati Y, Herrmann N, Swardfager W, Liu H, Sham L, Reim EK et al. A meta-analysis of cytokines in major depression. Biol Psychiatry 2010; 67: 446–457. 20015486 10.1016/j.biopsych.2009.09.033

[bib67] Liberto CM, Albrecht PJ, Herx LM, Yong VW, Levison SW. Pro-regenerative properties of cytokine-activated astrocytes. J Neurochem 2004; 89: 1092–1100. 15147501 10.1111/j.1471-4159.2004.02420.x

