# Supplementary material for: High-field magnetic resonance imaging of structural alterations in first-episode, drug-naive patients with major depressive disorder
Source: Transl Psychiatry. 2016 Nov 8;6(11):e942–. doi: 10.1038/tp.2016.209 (PMC5314121; doi:10.1038/tp.2016.209)
Supplement: Supplementary Table 1 [file tp2016209x1.docx]

**Supplementary Table 1.** Region-of-interest analysis of the magnetization transfer ratios between major depressive disorder patients and healthy subjects.

| Region-of-interest | Mean ± SD | | *p* value |
| --- | --- | --- | --- |
|  | MDD | HC |  |
| L Hippocampus | 36.70 ± 0.98 | 32.94 ± 1.61 | 0.449 |
| R Hippocampus | 32.71 ± 0.94 | 32.59 ± 2.88 | 0.859 |
| L Medial orbitofrontal | 34.46 ± 1.21 | 33.92 ± 1.92 | 0.186 |
| R Medial orbitofrontal | 33.73 ± 1.25 | 33.52 ± 1.95 | 0.628 |
| L Caudate nucleus | 32.70 ± 1.53 | 31.97 ± 5.11 | 0.489 |
| R Caudate nucleus | 31.70 ± 1.55 | 31.29 ± 4.51 | 0.675 |
| L Globus pallidus | 38.89 ± 0.98 | 37.48 ± 6.55 | 0.279 |
| R Globus pallidus | 39.30 ± 0.76 | 37.73 ± 7.13 | 0.273 |
| L Thalamus | 38.82 ± 0.95 | 38.40 ± 6.01 | 0.737 |
| R Thalamus | 38.10 ± 0.73 | 37.10 ± 5.25 | 0.343 |

Abbreviations: HC = healthy controls; L = left; MDD = major depressive disorder; R = right; SD = standard deviation.
